# Preliminary Evaluation of Recombinant EPC1 and TPx for Serological Diagnosis of Animal Cystic Echinococcosis

**DOI:** 10.3389/fcimb.2020.00177

**Published:** 2020-04-30

**Authors:** Yuqing Liang, Hongyu Song, Maodi Wu, Yue Xie, Xiaobin Gu, Ran He, Weiming Lai, Bo Jing, Xuerong Peng, Guangyou Yang

**Affiliations:** ^1^Department of Parasitology, College of Veterinary Medicine, Sichuan Agricultural University, Chengdu, China; ^2^Department of Chemistry, College of Life and Basic Science, Sichuan Agricultural University, Chengdu, China

**Keywords:** *Echinococcus granulosus*, serodiagnosis, diagnosis, thioredoxin peroxidase, indirect ELISA

## Abstract

Animal cystic echinococcosis (CE) is one of the most important helminthic diseases and affects many mammalian intermediate hosts. Practical and effective diagnosis is crucial for animal CE control. Two different recombinant antigens derived from *Echinococcus granulosus, Echinococcus* protoscolex calcium binding protein 1 (r*Eg*-EPC1) and thioredoxin peroxidase (r*Eg*-TPx), were evaluated in this study to detect the specific immunoglobulin G (IgG) in sheep and goat with CE by the indirect enzyme-linked immunosorbent assays. The diagnostic effect of the above-listed proteins was determined to their sensitivity and specificity and compared with hydatid cyst fluid, two previously reported immunogenic recombinant proteins (dihydrofolate reductase and P29), and two commercial kits available in China. Of these, the best diagnostic results were obtained in the anti-TPx IgG ELISA, with 92.6% sensitivity, 98.8% specificity, and no cross-reactivity with anti-*Eg*95 IgG. Recombinant *E. granulosus* thioredoxin peroxidase shows good potential for serological diagnosis of animal cystic echinococcosis.

## Introduction

Cystic echinococcosis (CE), caused by the larvae of *Echinococcus granulosus*, is a zoonotic disease included in the list of Neglected Tropical Diseases in the strategic plan of the World Health Organization (Eckert et al., 2001; Mcmanus et al., 2003). This zoonosis is characterized by long-term unilocular cyst growth in a wide range of domestic and wild animals, especially sheep, goats, and humans (Jenkins et al., 2005; Brunetti et al., 2011). CE has a global distribution in pastoral and rangeland areas such as the eastern part of the Mediterranean, northern Africa, eastern and southern Europe, southern South America, central Asia. CE is endemic in at least 368 counties in China (Qian et al., 2017). Worldwide, this zoonosis causes annual losses of about US$3 billion in livestock husbandry from liver condemnation, reduction in carcass weight, decrease in hide value, decrease in milk production, and reduced fertility (Oteroabad and Torgerson, 2013; Agudelo Higuita et al., 2017; Qian et al., 2017).

The detection of animal CE mainly relies on necropsy and molecular and serological diagnosis (Tamarozzi et al., 2014; Manzanoromán et al., 2015; Ito et al., 2016). For laboratory detection and surveillance, lab-based serology such as enzyme-linked immunosorbent assays (ELISAs) (Simsek and Koroglu, 2004), counter immunoelectrophoresis (Zhang and Mcmanus, 2013), latex agglutination (Jeyathilakan et al., 2014), and colloidal gold immunochromatographic strips (Zhuo et al., 2017) have been developed. However, because of the very long period of incubation and different organ localization, detection, and surveillance of CE infection is often inaccurate (error rate 15.4 %) (Gottstein, 1992; Gatti et al., 2007; Moro and Schantz, 2009). In particular, there has been limited study on recombinant proteins for CE detection in sheep and goat. Due to the great economic losses this disease causes, a sensitive and accurate serological diagnosis technique is required to help control CE (Reinhold et al., 2002; Zheng et al., 2013).

In this study, we compared serological diagnosis using two recombinant antigens (*Echinococcus* protoscolex calcium binding protein [r*Eg*-EPC1] and thioredoxin peroxidase [r*Eg*-TPx]) to detect *E. granulosus* in sheep and goat. TPx and EPC1 are of proven value in human CE as antigens (Li et al., 2003; Margutti et al., 2008). Sera from animals infected with the most common sheep and goat parasites were used to assess the cross-reactivity of these antigens. In addition, the protein with the best serodiagnostic potential was tested with the anti-*Eg*95 antibody. Furthermore, the tested antigens were compared with hydatid cyst fluid (HF), two previously reported recombinant immunogenic proteins (dihydrofolate reductase [r*Eg*-DHFR] and r*Eg*-P29) (Nouir et al., 2009; Song et al., 2017), and two commercial CE detection kits available in China. The study aimed to provide a sensitive and accurate detection method for CE to contribute to animal CE control.

## Materials and Methods

### Parasites

Cysts of *E. granulosus* (G1 genotype) were seperated from a naturally infected sheep from a slaughterhouse in Sichuan Province, China. The protoscolices (PSCs) were separated from individual fertile cysts, clarified by centrifugation at 600 × g for 5 min, and washed four times in phosphate-buffered saline (PBS) in sterile conditions as previously described (Song et al., 2016).

### Sera

Twenty-seven positive sera from naturally infected sheep and 24 negative sera from healthy sheep with no cysts were obtained in Sichuan Province. Serum samples from 64 goats infected with other parasites (21 *Cysticercus tenuicollis*, 20 *Taenia multiceps*, 16 *Haemonchus contortus*, and 7 *Fasciola hepatica*) and from 15 sheep infected with *Moniezia expansa* were also collected. All sera were determined by autopsy.

Sixty anti-*Eg*95 serum samples were collected from goats 1 month after immunization; 6-month-old goats were immunized twice under the skin, 1 month apart, with the immunological dose of 50 μg *Eg*95-GST protein plus 1 mg Quil-A adjuvant.

### Preparation of Antigens

Total RNA from PSCs was extracted using the RNA-prep Tissue Kit (Cowin Biotech, Beijing, China) and transcribed into cDNA by the Reverse Transcription System Kit (Thermo Fisher, USA) according to the manufacturers' instructions. Primers for PCR were designed based on transcriptome data (Table 1). The amplified PCR products were ligated into expression vector pET32a(+) (Novagen, Madison, WI) and transformed into *Escherichia coli* BL21 (DE3) (Cowin Biotech). Protein expression was induced with 0.1 mM isopropyl-β-D-thiogalactopyranoside (IPTG; Applied Biosystems, United Kingdom) at 37°C for 6 h with gentle shaking. Recombinant protein was purified from bacterial lysate using a Ni^2+^ affinity column (Bio-Rad, Hercules, CA) following the manufacturer's instructions. Crude HF antigen was extracted from hydatid cysts as previously described (Song et al., 2016). r*Eg*-DHFR and r*Eg*-P29 were provided by the Department of Parasitology of Sichuan Agricultural University.

**Table 1:** Primers used for PCR amplification of each antigen.

**Name**	**Primer (5^′^→ 3^′^)**	**Restriction site**
TPx-F	CGCGGATCCATGGCTGCTGTTGTTGG	*Bam*HI
TPx-R	CCGGAATTCTCACGAGCTCATGAACGA	*Eco*RI
EPC1-F	CGGGATCCATGAGTCTTCAGAAAACT	*Bam*HI
EPC1-R	CGGAATTCTTAGAAGAGAGCCATTAA	*Eco*RI

*The restriction site is underlined in each primer sequence*.

### Western Blotting

The recombinant proteins were separated by 12% SDS-PAGE and transferred onto nitrocellulose membranes. Blots were blocked with 5% skim milk at room temperature for 2 h after washing three times for 5 min each in Tris-buffered saline-Tween (TBST). The membranes with recombinant proteins were incubated with serum of infected sheep (1:200 v/v dilution) overnight at 4°C. The membranes were probed using 1:2,000-diluted horseradish peroxidase (HRP)-conjugated rabbit anti-sheep IgG (Bio-Rad) after washing and visualized using an enhanced HRP-DAB Chromogenic Substrate Kit (Tiangen, Beijing, China). Serum of healthy sheep was used for negative controls.

### Development of Indirect ELISA

The optimal conditions of antigens and serum were determined by a chessboard titration (Crowther, 2000). The proteins used as antigens were diluted in 0.1 M carbonate buffer (pH 9.6) and incubated overnight at 4°C in 96-well polystyrene plates. After washing with PBS (pH 7.4) and 0.05% Tween-20 three times for 5 min each wash, the plates were coated with 5% skim milk diluted in PBS (blocking buffer) at 37°C for 1 h. Serum samples (100 μl) were added to each plate and incubated at 37°C for 1 h. After the plates were washed as described above, HRP labeled rabbit anti-sheep/goat IgG (diluted 1:3,000 in PBS) was added into the plates (100 μl/well) at 37°C for 1 h. Finally, the reaction was developed in the dark with 100 μl/well of 3,3,5,5-tetramethylbenzidine (Tiangen) for 15 min and stopped by the addition of 100 μl 2 M H_2_SO_4_. The optical density (OD) of the wells was determined at 450 nm using an iELISA reader.

Twenty-four negative serum samples from healthy sheep were tested in the optimal conditions and the cut-off value was calculated as the mean OD_450_ plus three standard deviations of the OD_450_.

### Repeatability and Reproducibility of iELISA

The repeatability (intra-assay) and reproducibility (inter-assay) of the iELISA were tested using six serum samples positive against *E. granulosus*. With two groups set up, every sample was detected in one plate to assess the repeatability, and then tested consecutively in three plates to assess the reproducibility. After three repeats, the coefficient of variation (CV) was calculated.

### Sensitivity and Specificity

Sensitivity of the antigens was detected using 27 serum samples from *E. granulosus*-infected sheep. Specificity was determined using 64 serum samples from goat infected with other parasites: *C. tenuicollis* (*n* = 21), *T*. *multiceps* (*n* = 20), *H. contortus* (*n* = 16), and *F. hepatica* (*n* = 7) and from sheep infected with *M. expansa* (*n* = 15).

The analytical sensitivity percentage was calculated as ELISA positive ×100/true positive.

The analytical specificity percentage was calculated as ELISA negative ×100/true positive cross-reactivity.

### Detection of Anti-*Eg*95 Antibody

Cross-reactivity of test antigen with anti-*Eg*95 antibody was evaluated using serum from 60 immunized goats. After evaluating the sensitivity and specificity of the tested antigens as described above, the protein with the best serodiagnostic potential was detected with the anti-*Eg*95 antibody.

## Results

### Preparation of Recombinant Antigens

**Figure 1:**
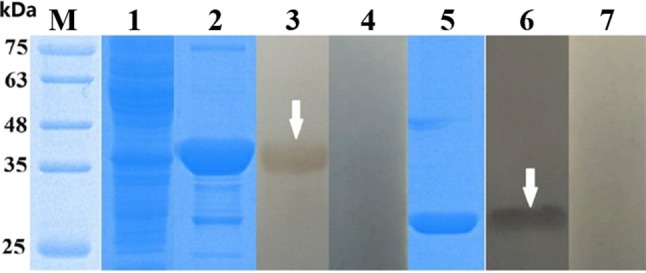
SDS-PAGE and western blotting of *Echinococcus granulosus* thioredoxin peroxidase (TPx) and EPC1. Lanes: M, protein molecular weight markers (in kDa); 1, empty vector pET32a(+) expressed in *Escherichia coli* BL21 (DE3); 2, purified rTPx; 3, purified r*Eg*-TPx detected using serum from a sheep naturally infected with *E. granulosus*; 4, purified r*Eg*-TPx tested with negative sheep serum; 5, purified EPC1; 6, purified r*Eg*-EPC1 detected using serum from a sheep naturally infected with *E. granulosus*; 7: purified r*Eg*-EPC1 tested with negative sheep serum. All sera used in western blotting were diluted 1:200 with 0.01 M phosphate-buffered saline.

The cDNA encoding *Eg*-EPC1 and *Eg*-TPx were obtained by PCR while the sequence of each was uploaded to NCBI (GenBank: MH603807, MH603808, respectively). After induction by 1 mM IPTG, SDS-PAGE showed that the His-tagged recombinant proteins of interest were expressed in *E. coli* BL21 cells. r*Eg*-TPx and r*Eg*-EPC1 were expressed as soluble proteins and purified by Ni^2+^ affinity chromatography (Figure 1, lanes 2 and 5).

### Western Blotting

The recombinant antigens were recognized by serum from sheep infected with *E. granulosus* (Figure 1, lanes 3 and 6), but not recognized by negative control serum (Figure 1, lanes 4 and 7), which suggested strong reactivity and good antigenicity of the recombinant proteins.

### ELISA

The optimal antigen concentrations and serum dilution were determined based on chessboard titration studies (Table 2). A total of 24 sheep sera were used to determine the OD_450_ cut-off value for each antigen (Table 2). The cut-off values were used as a standard for subsequent test results. Both the intra- and inter-assay variabilities were <10%.

**Table 2:** Comparative evaluation of serological assays for diagnosis of CE in sheep.

**Antigen**	**Optimal concentration of antigen**	**Optimal serum dilution**	**Cut-off value**	**Sensitivity**	**Specificity**
TPx	0.90 μg/well	1:40	0.792	92.6% (25/27)	98.8% (78/79)
EPC1	0.32 μg/well	1:40	0.759	100% (27/27)	49.4% (39/79)
P29	0.45 μg/well	1:40	0.731	88% (24/27)	89.9% (71/79)
DHFR	0.83 μg/well	1:80	0.464	88% (24/27)	88.6% (70/79)
HF	1:1,000 dilution	1:40	0.687	66.7% (18/27)	54.4% (43/79)
Kit A	NA	NA	NA	70.4% (19/27)	86.1% (68/79)
Kit B	NA	NA	NA	77.8% (21/27)	64.6% (51/79)

*A and B are commercial kits available in China. NA, not applicable. Details of the result of chessboard titration studies for each antigen are presented in Tables S1–S5*.

In the optimal conditions for each protein in indirect ELISA, detection of specific IgG in sera was performed using 27 positive samples from sheep infected with *E. granulosus*. The analytical sensitivities of r*Eg*-TPx, r*Eg*-EPC1, r*Eg*-P29, r*Eg*-DHFR, and HF were 92.6, 100, 88, 88, and 66.7%, respectively. The analytical specificity determined using 79 serum samples from sheep/goats infected with other parasites was 98.8, 49.4, 89.9, 88.6, and 54.4% for r*Eg*-TPx, r*Eg*-EPC1, r*Eg*-P29, r*Eg*-DHFR, and HF, respectively. The ELISA based on r*Eg*-TPx cross-reacted with only one serum sample from a sheep infected with another parasite (one *T. multiceps*-positive serum) (Table 3) and no cross-reactivity with anti-*Eg*95 antibody. Commercial kits A and B used following the manufacturer's guidelines showed sensitivity of 70.4 and 77.8%, and specificity of 86.1 and 64.6%, respectively (Table 2). These results indicated that ELISA based on *Eg*-TPx is practical and has potential for the diagnosis of CE.

**Table 3:** Comparative evaluation of the cross-reactivity of antigens.

	**Diagnosis positive/true positive**				
**Antigen/serum**	***M. expansa***	***C.tenuicollis***	***T. multiceps***	***H. contortus***	***F. hepatica***
TPx	0/15	0/21	1/20	0/16	0/7
EPC1	4/15	10/21	15/20	8/16	3/7
P29	2/15	2/21	2/20	1/16	1/7
DHFR	2/15	2/21	0/20	3/16	1/7

## Discussion

Cystic echinococcosis is a major zoonosis infecting humans and a wide range of animals, causing global health problems and economic losses worldwide (Sarkar et al., 2017). Diagnosis of CE by means of serology has limited support in clinical practice (Golassa et al., 2011; Jeyathilakan et al., 2014). Due to the poor specificity, complex composition and unstable antigen sources of natural proteins (Tamarozzi et al., 2014), establishing an accurate and sensitive immunologic diagnostic method based on recombinant protein has become crucial for CE detection.

In this context, we designed the present study to compare the diagnostic efficacy of two recombinant antigens—r*Eg*-EPC1 and r*Eg*-TPx—in the detection of sera from sheep infected with CE. Some authors advocate the use of purified hydatid cyst fluid antigens in serological CE detection (Rogan et al., 1991; Wen and Craig, 1994; Elon et al., 1997). However, in the present study, with a cut-off value of 0.687, the HF showed diagnostic sensitivity of only 66.7% (18/27) and specificity of 54.4% (43/79). Commercial kits are widely used for detecting CE because they are rapid and cheap. However, the two commercial kits we tested in this study only showed sensitivity of 70.4 and 77.8%, and specificity of 86.1 and 64.6%, respectively. These results indicate the inaccuracy of HF and commercial kits for CE detection.

EPC1 is a calcium-binding protein with great immunogenicity. In previous study, recombinant EPC1 showed sensitivity of 92.2% and specificity of 95.6% in immunoblotting in detection of CE patient serum (Li et al., 2003). In contrast, recombinant EPC1 reacted fairly with sera from infected goats and buffaloes with sensitivity of 60 and 89.3%, respectively, meanwhile with specificity of 72.3% and 51.5% (Rialch et al., 2017, 2018). P-29, a 29-kDa antigen from *E. granulosus*, was first described by González Muzio et al. (2000); subsequently, recombinant P29 has been used as a biomarker for post-therapy CE patients (Margutti et al., 2008). Dihydrofolate reductases (DHFRs) are ubiquitous enzymes vital for purine and thymidylate synthesis in cells (Jennewein and Zmasek, 2013). In previous study, Song et al. (2017) identified the serological diagnostic potential of recombinant DHFR, which had specificity of 89.58%, diagnostic sensitivity of 95.83%, and diagnostic accuracy of 91.67% in iELISA detection. Here, recombinant EPC1 exhibited poor specificity (49.4%) in detection of CE in sheep, indicating that rEPC1 is not suitable for ovine CE immunodiagnosis. Meanwhile, rP29 and rDHFR were less effective in serodiagnosis (sensitivity 88 and 88%, specificity 89.9 and 88.6%, respectively) than rTPx (sensitivity 92.6%, specificity 98.8%).

TPx was first identified in *Saccharomyces cerevisiae* (Kim et al., 1989), and is widely distributed in both helminthic and protozoan parasites (Kim et al., 2000). Its main function is to remove reactive oxygen species and H_2_O_2_ derived from normal cellular metabolism (Tsuji et al., 2000; Henkle-Dührsen and Kampkötter, 2001). Li et al. (2004) suggested that *Eg*-TPx may be a target for hydatid disease chemotherapy. Previous work has shown great diagnostic value of TPxs, for instance of *Fasciola gigantica*-TPx (Zhang et al., 2011) and *Schistosoma japonicum-*TPx (Angeles et al., 2012). In previous study, rTPx showed sensitivity of 83% and specificity of 92% in detecting CE in patient serum by iELISA assay (Margutti et al., 2008), suggesting that TPx could be a candidate for CE immunodiagnosis. In the present study, recombinant *Eg*-TPx protein showed great immunoreactivity and diagnostic potential with a cut-off value of 0.729 in indirect ELISA using sheep serum positive for *E. granulosus*. Compared with the other tested antigens and two commercial kits, TPx displayed a better diagnosis performance in CE-infected sera; the sensitivity was 92.6%, the specificity was 98.8%, and no cross-reactivity was observed with anti-*Eg*95 IgG. The high sensitivity and specificity of rTPx in our study might relate to its different protein structure and more epitopes compared to other tested antigens. In addition, the antibody titers could be another reason to explain the sensitivity and specificity differences which vary with host species, CE loads and the period of infection. The newly established indirect ELISA relying on r*Eg*-TPx shows great value for CE detection in sheep, facilitating the pre-inspection process and subsequent infection confirmation and control.

This paper was conducted on a smaller number of sera samples but screening of larger number of both the cross-reactivity and positive samples of sheep and goats is needed for validating the utility of rEg-EPC1 and rEg-TPx in the sero-diagnosis of cystic echinococcosis in animals.

## Data Availability Statement

The datasets generated for this study can be found in NCBI, accession numbers MH603807, MH603808.

## Ethics Statement

The animal study was reviewed and approved by the Animal Care and Use Committee of Sichuan Agricultural University (SYXK2019-187). All animal procedures used in this study were carried out in accordance with the guide for the Care and Use of Laboratory Animals (National Research Council, Bethesda, MD, USA) and recommendations of the ARRIVE guidelines (http://www.nc3rs.org.uk/arrive-guidelines). All methods were carried out in accordance with relevant guidelines and regulations.

## Author Contributions

YL and HS performed most of the study, analyzed the data, and contributed to manuscript writing. MW, YX, XG, RH, and WL provided technical assistance. BJ and XP contributed to partial ELISA study and discussion. GY conceived and designed the study plan, participated in all aspects of the study, provided funds, and supervised the research. All authors read and approved the final manuscript.

## Conflict of Interest

The authors declare that the research was conducted in the absence of any commercial or financial relationships that could be construed as a potential conflict of interest.
