# Supplementary material for: Preliminary Evaluation of Recombinant EPC1 and TPx for Serological Diagnosis of Animal Cystic Echinococcosis
Source: Front Cell Infect Microbiol. 2020 Apr 30;10:177. doi: 10.3389/fcimb.2020.00177 (PMC7203340; doi:10.3389/fcimb.2020.00177)
Supplement: Supplementary file 1 [file Data_Sheet_1.docx]

**Journal name: Frontiers in Cellular and Infection Microbiology**

**Preliminary evaluation of recombinant EPC1 and TPx for serological diagnosis of animal cystic echinococcosis**

Yuqing Liang^1^†, Hongyu Song^1^†, Maodi Wu^1^, Yue Xie^1^, Xiaobin Gu^1^, Ran He^1^, Weiming Lai^1^, Bo Jing^1^, Xuerong Peng ^2^ and Guangyou Yang ^1^*

^1^Department of Parasitology, College of Veterinary Medicine, Sichuan Agricultural University, Chengdu 611130, China

^2^Department of Chemistry, College of Life and Basic Science, Sichuan Agricultural University, Chengdu 611130, China

Corresponding author: GuangYou Yang

Email: [guangyou1963@aliyun.com](mailto:guangyouy1963@aliyun.com)

Telephone number: +86-18980558366

Tax number: 0086-028-82652669

**TableS1 Determination of the optimal antigen r*Eg*-TPx concentration and serum dilution**

| **Antigen concentration**  **μg/well** | | **Serum dilution** | | | | | |
| --- | --- | --- | --- | --- | --- | --- | --- |
|  |  | 1:20 1:40 1:80 1:160 1:320 1:640 | | | | | |
| 28.8 | P | 1.432 | 1.221 | 0.868 | 0.668 | 0.521 | 0.395 |
|  | N | 0.685 | 0.553 | 0.495 | 0.400 | 0.322 | 0.276 |
|  | P/N | 2.091 | 2.208 | 1.754 | 1.670 | 1.618 | 1.431 |
|  | | | | | | | |
| 14.4 | P | 1.343 | 1.216 | 0.866 | 0.661 | 0.502 | 0.386 |
|  | N | 0.581 | 0.517 | 0.487 | 0.392 | 0.318 | 0.263 |
|  | P/N | 2.312 | 2.352 | 1.778 | 1.686 | 1.579 | 1.468 |
|  | | | | | | | |
| 7.2 | P | 1.297 | 1.210 | 0.864 | 0.655 | 0.505 | 0.377 |
|  | N | 0.562 | 0.511 | 0.472 | 0.384 | 0.315 | 0.255 |
|  | P/N | 2.308 | 2.368 | 1.831 | 1.706 | 1.603 | 1.478 |
|  | | | | | | | |
| 3.6 | P | 1.195 | 1.190 | 0.799 | 0.625 | 0.470 | 0.364 |
|  | N | 0.524 | 0.498 | 0.465 | 0.374 | 0.302 | 0.250 |
|  | P/N | 2.280 | 2.390 | 1.718 | 1.671 | 1.556 | 1.456 |
|  | | | | | | | |
| 1.8 | P | 1.056 | 1.115 | 0.846 | 0.641 | 0.468 | 0.371 |
|  | N | 0.467 | 0.438 | 0.481 | 0.372 | 0.298 | 0.245 |
|  | P/N | 2.261 | 2.546 | 1.759 | 1.723 | 1.570 | 1.514 |
|  | | | | | | | |
| 0.9 | P | 1.189 | ***1.115*** | 0.846 | 0.641 | 0.468 | 0.371 |
|  | N | 0.568 | ***0.436*** | 0.479 | 0.362 | 0.301 | 0.239 |
|  | P/N | 2.093 | ***2.587*** | 1.739 | 1.765 | 1.525 | 1.552 |
|  | | | | | | | |
| 0.45 | P | 1.012 | 0.997 | 0.832 | 0.634 | 0.443 | 0.366 |
|  | N | 0.493 | 0.410 | 0.476 | 0.354 | 0.297 | 0.235 |
|  | P/N | 2.053 | 2.432 | 1.748 | 1.791 | 1.492 | 1.557 |
|  | | | | | | | |
| 0.23 | P | 1.005 | 0.988 | 0.822 | 0.629 | 0.438 | 0.355 |
|  | N | 0.454 | 0.407 | 0.464 | 0.343 | 0.267 | 0.231 |
|  | P/N | 2.214 | 2.428 | 1.772 | 1.834 | 1.640 | 1.537 |

Note: The optimal antigen concentration and serum dilution was in a bond, italic, underlined font. P:positive serum N:negative serum

**TableS2 Determination of the optimal antigen r*Eg*-EPC1 concentration and serum dilution**

| **antigen**  **concentration**  **μg/well** | | **serum dilution** | | | | | |
| --- | --- | --- | --- | --- | --- | --- | --- |
|  |  | 1:20 | 1:40 | 1:80 | 1:160 | 1:320 | 1:640 |
| 20.48 | P | 1.300 | 1.017 | 0.785 | 0.554 | 0.391 | 0.293 |
|  | N | 0.405 | 0.364 | 0.294 | 0.251 | 0.220 | 0.198 |
|  | P/N | 3.210 | 2.794 | 2.670 | 2.207 | 1.777 | 1.480 |
|  |  |  |  |  |  |  |  |
| 10.24 | P | 1.227 | 0.932 | 0.729 | 0.505 | 0.358 | 0.263 |
|  | N | 0.391 | 0.311 | 0.281 | 0.207 | 0.270 | 0.165 |
|  | P/N | 3.138 | 2.997 | 2.594 | 2.440 | 1.326 | 1.594 |
|  |  |  |  |  |  |  |  |
| 5.12 | P | 1.118 | 0.932 | 0.644 | 0.485 | 0.322 | 0.234 |
|  | N | 0.372 | 0.303 | 0.274 | 0.213 | 0.188 | 0.167 |
|  | P/N | 3.005 | 3.076 | 2.350 | 2.277 | 1.713 | 1.401 |
|  |  |  |  |  |  |  |  |
| 2.56 | P | 1.064 | 0.845 | 0.615 | 0.450 | 0.324 | 0.236 |
|  | N | 0.351 | 0.314 | 0.246 | 0.205 | 0.179 | 0.181 |
|  | P/N | 3.031 | 2.691 | 2.500 | 2.195 | 1.810 | 1.304 |
|  |  |  |  |  |  |  |  |
| 1.28 | P | 0.930 | 0.792 | 0.586 | 0.419 | 0.302 | 0.236 |
|  | N | 0.369 | 0.285 | 0.231 | 0.205 | 0.164 | 0.160 |
|  | P/N | 2.520 | 2.779 | 2.537 | 2.044 | 1.841 | 1.475 |
|  |  |  |  |  |  |  |  |
| 0.64 | P | 0.924 | 0.833 | 0.590 | 0.406 | 0.310 | 0.218 |
|  | N | 0.363 | 0.282 | 0.238 | 0.200 | 0.170 | 0.160 |
|  | P/N | 2.545 | 2.954 | 2.479 | 2.030 | 1.824 | 1.363 |
|  |  |  |  |  |  |  |  |
| 0.32 | P | 0.961 | ***0.829*** | 0.586 | 0.399 | 0.293 | 0.213 |
|  | N | 0.352 | ***0.249*** | 0.215 | 0.183 | 0.163 | 0.154 |
|  | P/N | 2.730 | ***3.329*** | 2.726 | 2.180 | 1.798 | 1.383 |
|  |  |  |  |  |  |  |  |
| 0.16 | P | 0.967 | 0.724 | 0.528 | 0.390 | 0.279 | 0.211 |
|  | N | 0.366 | 0.294 | 0.249 | 0.206 | 0.170 | 0.180 |
|  | P/N | 2.642 | 2.463 | 2.120 | 1.893 | 1.641 | 1.172 |

Note: The optimal antigen concentration and serum dilution was in a bond, italic, underlined font. P:positive serum N:negative serum

**TableS3 Determination of the optimal antigen r*Eg*-P29concentration and serum dilution**

| **antigen concentration**  **μg/well** | | **serum dilution** | | | | | |
| --- | --- | --- | --- | --- | --- | --- | --- |
|  |  | 1:20 | 1:40 | 1:80 | 1:160 | 1:320 | 1:640 |
| 14.4 | P | 1.173 | 0.998 | 0.842 | 0.690 | 0.540 | 0.409 |
|  | N | 0.503 | 0.419 | 0.305 | 0.277 | 0.251 | 0.256 |
|  | P/N | 2.332 | 2.382 | 2.761 | 2.292 | 2.045 | 1.425 |
|  | | | | | | | |
| 7.2 | P | 1.140 | 0.995 | 0.797 | 0.630 | 0.503 | 0.382 |
|  | N | 0.439 | 0.388 | 0.327 | 0.277 | 0.251 | 0.256 |
|  | P/N | 2.597 | 2.564 | 2.437 | 2.274 | 2.004 | 1.492 |
|  | | | | | | | |
| 3.6 | P | 1.121 | 0.919 | 0.770 | 0.605 | 0.488 | 0.351 |
|  | N | 0.420 | 0.329 | 0.308 | 0.266 | 0.239 | 0.251 |
|  | P/N | 2.669 | 2.793 | 2.500 | 2.274 | 2.042 | 1.398 |
|  | | | | | | | |
| 1.8 | P | 1.044 | 0.903 | 0.728 | 0.593 | 0.450 | 0.349 |
|  | N | 0.401 | 0.332 | 0.291 | 0.250 | 0.227 | 0.228 |
|  | P/N | 2.603 | 2.720 | 2.502 | 2.372 | 1.982 | 1.531 |
|  | | | | | | | |
| 0.90 | P | 0.988 | 0.836 | 0.685 | 0.525 | 0.409 | 0.313 |
|  | N | 0.340 | 0.291 | 0.259 | 0.222 | 0.204 | 0.192 |
|  | P/N | 2.906 | 2.873 | 2.645 | 2.365 | 2.005 | 1.630 |
|  | | | | | | | |
| 0.45 | P | 0.867 | ***0.762*** | 0.623 | 0.474 | 0.348 | 0.68 |
|  | N | 0.302 | ***0.255*** | 0.245 | 0.208 | 0.187 | 0.184 |
|  | P/N | 2.871 | ***2.988*** | 2.543 | 2.279 | 1.861 | 1.457 |
|  | | | | | | | |
| 0.23 | P | 0.794 | 0.657 | 0.542 | 0.403 | 0.304 | 0.243 |
|  | N | 0.278 | 0.238 | 0.216 | 0.185 | 0.174 | 0.166 |
|  | P/N | 2.856 | 2.761 | 2.509 | 2.178 | 1.747 | 1.464 |
|  | | | | | | | |
| 0.12 | P | 0.747 | 0.646 | 0.463 | 0.351 | 0.271 | 0.218 |
|  | N | 0.280 | 0.230 | 0.207 | 0.180 | 0.167 | 0.164 |
|  | P/N | 2.668 | 2.722 | 2.237 | 1.950 | 1.623 | 1.329 |

Note: The optimal antigen concentration and serum dilution was in a bond, italic, underlined font. P:positive serum N:negative serum

**TableS4 Determination of the optimal antigen r*Eg*-DHFR concentration and serum dilution**

| **antigen concentration**  **μg/well** | | **serum dilution** | | | | | |
| --- | --- | --- | --- | --- | --- | --- | --- |
|  |  | 1:20 | 1:40 | 1:80 | 1:160 | 1:320 | 1:640 |
| 53.12 | P | 1.428 | 1.298 | 1.082 | 0.977 | 0.738 | 0.527 |
|  | N | 0.942 | 0.739 | 0.586 | 0.469 | 0.393 | 0.343 |
|  | P/N | 1.516 | 1.602 | 1.712 | 1.998 | 1.693 | 1.324 |
|  |  |  |  |  |  |  |  |
| 26.56 | P | 1.389 | 1.278 | 1.067 | 0.870 | 0.703 | 0.525 |
|  | N | 0.936 | 0.739 | 0.586 | 0.469 | 0.393 | 0.343 |
|  | P/N | 1.484 | 1.729 | 1.821 | 1.855 | 1.789 | 1.531 |
|  |  |  |  |  |  |  |  |
| 13.28 | P | 1.330 | 1.210 | 1.002 | 0.857 | 0.662 | 0.508 |
|  | N | 0.817 | 0.710 | 0.565 | 0.465 | 0.378 | 0.349 |
|  | P/N | 1.628 | 1.704 | 1.773 | 1.843 | 1.751 | 1.456 |
|  |  |  |  |  |  |  |  |
| 6.64 | P | 1.297 | 1.108 | 0.887 | 0.717 | 0.592 | 0.433 |
|  | N | 0.689 | 0.605 | 0.488 | 0.408 | 0.337 | 0.312 |
|  | P/N | 1.882 | 1.831 | 1.818 | 1.757 | 1.757 | 1.388 |
|  |  |  |  |  |  |  |  |
| 3.32 | P | 1.197 | 1.031 | 0.803 | 0.634 | 0.494 | 0.373 |
|  | N | 0.573 | 0.508 | 0.420 | 0.346 | 0.289 | 0.278 |
|  | P/N | 2.089 | 2.030 | 1.912 | 1.832 | 1.709 | 1.342 |
|  |  |  |  |  |  |  |  |
| 1.66 | P | 1.154 | 0.999 | 0.742 | 0.549 | 0.419 | 0.306 |
|  | N | 0.538 | 0.438 | 0.360 | 0.284 | 0.253 | 0.235 |
|  | P/N | 2.145 | 2.281 | 2.061 | 1.933 | 1.656 | 1.32 |
|  |  |  |  |  |  |  |  |
| 0.83 | P | 0.975 | 0.924 | ***0.744*** | 0.477 | 0.354 | 0.253 |
|  | N | 0.483 | 0.402 | ***0.317*** | 0.240 | 0.215 | 0.205 |
|  | P/N | 2.019 | 2.299 | ***2.347*** | 1.988 | 1.647 | 1.234 |
|  |  |  |  |  |  |  |  |
| 0.41 | P | 0.904 | 0.864 | 0.480 | 0.459 | 0.336 | 0.258 |
|  | N | 0.458 | 0.369 | 0.305 | 0.231 | 0.208 | 0.193 |
|  | P/N | 1.974 | 2.341 | 1.574 | 1.987 | 1.615 | 1.337 |

Note: The optimal antigen concentration and serum dilution was in a bond, italic, underlined font. P:positive serum N:negative serum

**TableS5 The OD_450_ of r*Eg*-TPx developed ELISA with anti-Eg95 antibody**

| **The OD_450_ of r*Eg*-TPx developed ELISA with anti-Eg95 antibody** | | | | | | | | | |
| --- | --- | --- | --- | --- | --- | --- | --- | --- | --- |
| 0.438 | 0.356 | 0.352 | 0.458 | 0.425 | 0.325 | 0.556 | 0.358 | 0351 | 0.512 |
| 0.432 | 0.362 | 0.456 | 0.412 | 0.512 | 0.453 | 0.546 | 0.487 | 0.465 | 0.398 |
| 0.356 | 0.546 | 0.462 | 0.356 | 0.365 | 0.522 | 0.458 | 0.501 | 0.425 | 0.354 |
| 0423 | 0.362 | 0.412 | 0.362 | 0.415 | 0.342 | 0.398 | 0.471 | 0.575 | 0.361 |
| 0.312 | 0.413 | 0.456 | 0.444 | 0.433 | 0.334 | 0.395 | 0.421 | 0.426 | 0.366 |
| 0.561 | 0.403 | 0.463 | 0.556 | 0.390 | 0.402 | 0.436 | 0.345 | 0.515 | 0.395 |
